# Genetic Diversity under Soil Compaction in Wheat: Root Number as a Promising Trait for Early Plant Vigor

**DOI:** 10.3389/fpls.2017.00420

**Published:** 2017-03-28

**Authors:** Tino Colombi, Achim Walter

**Affiliations:** Institute of Agricultural Sciences ETH ZurichZurich, Switzerland

**Keywords:** genetic diversity, phenotyping, mechanical impedance, root-shoot synchronization, soil compaction, X-ray computed tomography

## Abstract

Soil compaction of arable land, caused by heavy machinery constitutes a major threat to agricultural soils in industrialized countries. The degradation of soil structure due to compaction leads to decreased (macro-) porosity resulting in increased mechanical impedance, which adversely affects root growth and crop productivity. New crop cultivars, with root systems that are adapted to conditions of increased soil strength, are needed to overcome the limiting effects of soil compaction on plant growth. This study aimed (i) to quantify the genetic diversity of early root system development in wheat and to relate this to shoot development under different soil bulk densities and (ii) to test whether root numbers are suitable traits to assess the genotypic tolerance to soil compaction. Fourteen wheat genotypes were grown for 3 weeks in a growth chamber under low (1.3 g cm^-3^), moderate (1.45 g cm^-3^), and high soil bulk density (1.6 g cm^-3^). Using X-ray computed tomography root system development was quantified in weekly intervals, which was complemented by weekly measurements of plant height. The development of the root system, quantified via the number of axial and lateral roots was strongly correlated (0.78 < *r* < 0.88, *p* < 0.01) to the development of plant height. Furthermore, significant effects (*p* < 0.01) of the genotype on root system development and plant vigor traits were observed. Under moderate soil strength final axial and lateral root numbers were significantly correlated (0.57 < *r* < 0.84, *p* < 0.05) to shoot dry weight. Furthermore, broad-sense heritability of axial and lateral root number was higher than 50% and comparable to values calculated for shoot traits. Our results showed that there is genetic diversity in wheat with respect to root system responses to increased soil strength and that root numbers are suitable indicators to explain the responses and the tolerance to such conditions. Since root numbers are heritable and can be assessed at high throughput rates under laboratory and field conditions, root number is considered a promising trait for screening toward compaction tolerant varieties.

## Introduction

It is estimated that an area of 68 million hectares of arable land is degraded by soil compaction (Hamza and Anderson, 2005; Batey, 2009), which is caused by the increasing use of heavy agricultural machinery in modern agriculture (Tracy et al., 2011). In comparison to undisturbed soils, compacted soils are characterized by lower (macro-) porosity and decreased pore connectivity (Bottinelli et al., 2014; Chen G. et al., 2014; Kuncoro et al., 2014b). This soil structural degradation adversely affects soil physical functions, which limit root growth and therefore decrease agricultural productivity (Barraclough and Weir, 1988; Botta et al., 2010; Arvidsson et al., 2014). Decreased void space in compacted soils leads to higher mechanical impedance and hence results in reduced root growth rates and resource uptake (Bengough and Mullins, 1991; Young et al., 1997; Jin et al., 2013). Furthermore, crop growth in compacted soils may be limited due to low levels of plant available water (Lipiec and Hatano, 2003) and decreased fluid transport rates (Kuncoro et al., 2014a; Colombi et al., 2017). Together these adverse changes of soil physical functions lead to decreased physical soil fertility caused by soil compaction (Abbott and Murphy, 2007). High soil strength may also occur in dry soils, which are also characterized by increased mechanical impedance (Masle and Passioura, 1987; Bengough et al., 2011). Since high soil strength reduces primarily root system vigor, varieties with adapted root system traits are needed in order to overcome adverse effects of soil compaction on crop productivity. The integration of root traits into breeding programs is suggested to increase the tolerance of crops to soil derived abiotic stress and therefore to contribute to crop productivity under limited soil fertility (York et al., 2013).

In recent years, the concept of adapting the root system architecture of crops in a way that allows improving crop productivity under poor soil fertility received growing attention (Bishopp and Lynch, 2015). Root system architecture describes the spatial configuration of coarse structures of the root system based on the quantification of root numbers, lateral branching density and root angles in soil (Lynch, 1995). Among other root architectural properties, root numbers were shown to be related in different crops to the genotypic tolerance to low soil fertility. In maize for example, low axial and lateral root number were observed to enhance plant performance under conditions of low soil nitrogen (Saengwilai et al., 2014; Zhan and Lynch, 2015) and under low soil moisture (Zhan et al., 2015; Gao and Lynch, 2016). In common bean instead, a high number of basal roots in the top soil improved phosphorus uptake and plant vigor in low phosphorus soils (Miguel et al., 2013). A major advantage of root system architectural traits and root numbers in particular is that they can be assessed in large diversity panels under field conditions at high throughput rates (Trachsel et al., 2011; Colombi et al., 2015; Burridge et al., 2016). The quantification of root dry weight or length instead is much more laborious and not feasible under field conditions. The heritability of root numbers was reported to be relatively high in a wide range of crop species (Wilcox and Farmer, 1968; Bucksch et al., 2014; Colombi et al., 2015; Li et al., 2015; Richard et al., 2015; Burridge et al., 2016). Besides increasing awareness about the importance of roots for crop production, it has been suggested that holistic phenotyping approaches are needed to understand plant responses to abiotic stress. This may include simultaneous assessments of above and below ground traits and continuous measurements of plant traits instead of measurements taken at one single point in time (Walter et al., 2015).

Like other abiotic stresses such as water or nutrient scarcity, soil compaction causes alterations of the root system phenotype. These phenotypic responses are consistent between different crop species including major mono- and dicotyledonous crops such as small grain cereals, maize or soybean (Tracy et al., 2012; Chen Y.L. et al., 2014; Grzesiak et al., 2014; Pfeifer et al., 2014; Colombi and Walter, 2016). Apart from shallower root growth and increased root diameters, crop root systems show decreased axial and lateral root numbers in response to soil compaction. In most of these studies such alterations of the root phenotype resulted in decreased shoot biomass both under laboratory (Grzesiak et al., 2014; Pfeifer et al., 2014) and field conditions (Chen Y.L. et al., 2014; Colombi and Walter, 2016). It has been shown that these root architectural responses to soil compaction obtained from mature plants in the field can be reproduced in pots with young plants (Colombi and Walter, 2016). In the same study it was also observed that lateral root initiation in wheat, triticale, and soybean seedlings is delayed due to increased soil strength. However, in this study only one variety per crop species was assessed and thus no conclusive statement could be made about genetic diversity of plant responses to soil compaction within one species. Leaf and root growth rates of wheat, barley, maize, and pea were reported to decrease within minutes to hours when soil strength increased (Masle and Passioura, 1987; Bengough and Mullins, 1991; Beemster et al., 1996; Young et al., 1997). The susceptibility to increased soil strength varies considerably between different species. It has been shown that legumes are more sensitive to soil compaction than grasses (Arvidsson and Håkansson, 2014) and that small grain cereals show a higher tolerance to increased mechanical impedance than maize (Grzesiak et al., 2014).

Despite the information about phenotypic responses of crops to soil compaction and the differences between species, information about differences within a single species is scarce, but would be highly desired for plant breeding purposes. A detailed understanding of the genotypic diversity is needed to identify root traits, which determine the tolerance to increased soil strength (Hamza and Anderson, 2005). This includes the understanding of root-shoot relationships in compacted soils as well as quantitative information about root traits, which determine growth responses and the tolerance to increased soil strength. Genotypic differences of axial and lateral root number and shoot vigor were shown in young triticale, maize, and soybean plants. Depending on the genotype, moderate soil compaction in particular led to decreased, constant or even increased root numbers (Bushamuka and Zobel, 1998; Grzesiak et al., 2014). However, in these studies only two to four genotypes were evaluated, which did not allow for quantitative statements about the influence of root traits on the tolerance to soil compaction. In other studies soil compaction was simulated with paraffin-Vaseline disks and the capability to penetrate these disks was compared between seedlings of 24 and 81 wheat genotypes (Kubo et al., 2004, 2006). These studies reported a positive relationship between the number of roots penetrating through the paraffin-Vaseline layer and shoot dry weight. Similar results were reported for eight varieties of narrow-leafed lupin grown in the field, where the number of lateral roots was positively correlated to the agronomic performance of the different cultivars (Chen Y.L. et al., 2014). These studies indicated the use of certain root system architectural traits in order to increase the tolerance to soil compaction. However, due to the destructive measurements, root-shoot relationships could not be quantified dynamically in any of these studies. X-ray computed tomography or magnetic resonance imaging are promising approaches to study root system development in soil over time. Using X-ray micro computed tomography temporal dynamics of root system architecture in response to soil compaction were quantified in tomato, wheat, and soybean seedlings (Tracy et al., 2012, 2013; Colombi and Walter, 2016).

In this study we test the hypothesis whether root number is a suitable trait that could be used in crop breeding programs aiming to improve the tolerance to compacted soils by: (i) investigating how root system development is related to shoot development under increasing soil strength, (ii) quantifying the genetic diversity of root and shoot responses to increased soil strength, and (iii) testing whether root numbers may be used to assess the tolerance of different wheat varieties to increased soil strength. Fourteen wheat varieties were grown under three different levels of soil bulk density for 3 weeks, during which root system development and shoot growth were quantified in weekly intervals.

## Materials and Methods

### Plant Material and Soil Physical Conditions

The 14 winter wheat (*Triticum aestivum* L.) varieties used in this study, originate from Swiss public breeding programs and were released to the market between 1910 and 2010 (**Table 1**). Plants were grown in PVC columns of 4.9 cm inner diameter and 15 cm height, which were filled with field soil (Pseudogleyed Cambisol) excavated at Agroscope Zurich (8°31′E, 47°27′N, 443 m above sea level). For the experiments, soil was taken from the uppermost 15 cm, dried to approximately 22% gravimetric water content and homogenized before being sieved through a 2 mm sieve. Soil pH (CaCl_2_) in the top 20 cm was 6.9 with an organic carbon content of 1.7% and textural composition of 25% clay, 50% silt and 25% sand. The soil was stored at 3°C until further use. Different levels of soil strength were achieved by compressing the soil to three different soil bulk densities. Soil was packed into the columns to low (1.3 g cm^-3^), moderate (1.45 g cm^-3^), and high (1.6 g cm^-3^) bulk density in six layers of 2 cm height. In order to ensure homogenous packaging surfaces of each layer were slightly abraded. To ensure proper soil aeration, the columns were closed at the bottom with sheep wool. Each variety-bulk density combination was replicated four times.

**Table 1:** Winter wheat varieties used in the study ordered according to the year of market release.

Variety name	Year of release
Plantahof	1910
Mont-Calme 245	1926
Mont-Calme 268	1926
Probus	1948
Zenith	1969
Arina	1981
Runal	1995
Titlis	1996
Zinal	2003
CH-Claro	2007
Forel	2007
CH-Combin	2008
Suretta	2009
Simano	2010


Four individual soil cores of 5.1 cm diameter and 5 cm height per bulk density were packed with the same sieved soil as used for plant growth studies in 1 cm layers to low (1.3 g cm^-3^), moderate (1.45 g cm^-3^), and high (1.6 g cm^-3^) soil bulk density. These samples were saturated slowly from below and equilibrated on a ceramic plate to a matric potential of -100 hPa in order to determine gravimetric water content at field capacity (Schjonning and Rasmussen, 2000). Mechanical impedance at -100 hPa was determined by two penetrometer insertions into the center of the bottom side of the soil cores as described by Colombi and Walter (2016). Measured penetration resistance was 0.34 (±0.08 SD) MPa, 0.44 (±0.03 SD) MPa, and 1.06 (±0.22 SD) MPa for low, moderate and high bulk density, respectively.

### Growth Conditions

Pre-germinated seeds (25°C, 48 h) were selected according to similar radicle length and seed size. The emerged radicle (length ≈ 2 mm) was placed into a hole of 1 mm diameter and 5 mm length, which was inserted into the center of the soil columns. Seeds were covered with 1 cm of loose soil (1.0 g cm^-3^). Plants were allocated in a randomized complete block design with four blocks and grown for 23 days in a growth chamber at a day/night cycle of 14/10 h. Incident light was 510 (±33, SD) μmol s^-1^ m^-2^ and recorded average temperature and relative air humidity were 21.4°C and 63%, respectively. Soil moisture content was kept at field capacity (-100 hPa) by daily weighing and watering.

### Root and Shoot Growth Dynamics

**FIGURE 1:**
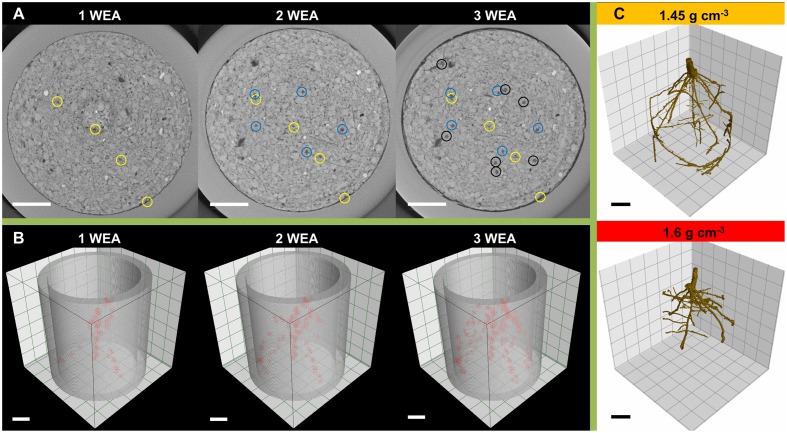
**Illustration of root system quantification by means of X-ray computed tomography:** **(A)** cross sections taken 1 cm below seed base 1, 2, and 3 weeks after shoot emergence (WAE) with axial roots marked (circles); **(B)** three dimensional arrangement of lateral branching points (red markers) within the top 6 cm of the soil column 1, 2, and 3 WAE; **(C)** segmentation of root system 2 WAE under moderate (1.45 g cm^-3^) and high soil bulk density (1.6 g cm^-3^); Scale bar = 1 cm.

Both root and shoot development was recorded four times during growth. This was done in weekly intervals, starting at leaf emergence, which occurred in all plants 2 days after planting. Similar to previous studies (Tracy et al., 2012, 2013; Colombi and Walter, 2016) root system development was quantified using an X-Ray micro computed tomography (μCT) scanner (Phoenix v| tome| x s 240; GE Sensing and Inspection Technologies GmbH, Wunstorf, Germany). The top 6 cm of the pot were scanned at 120 kV and 450 μA with 0.1 mm Cu filter. To reduce noise in the scans and scanning time binning of 2^∗^2 voxels was applied. The used settings (Supplementary Table 1) resulted in a scan time of 7 min and a voxel edge length of 0.068 mm. The number of axial and lateral roots were manually counted in each scan using Visual Studio Max 2.2 (Volume Graphics GmbH, Heidelberg, Germany) in a segment of 4 cm height, starting 1 cm below the seed base. Axial roots were only counted if they penetrated deeper than 1 cm below the seed base and lateral roots were counted if they emerged from axial roots at depths between 1 and 5 cm below the seed base (**Figure 1**). Visual detection limits for structures in μCT scans are commonly seen to be at diameters that exceed twice the voxel edge length (Jähne, 2002). Hence, the chosen resolution of 0.068 mm voxel edge length enabled to quantify first order lateral roots. The first time point 2 days after sowing was only used to confirm that roots successfully penetrated the soil and was excluded from further analysis. At the same days at which μCT scans were performed, shoot development was determined. These measurements included the number of fully developed leaves, the number of tillers and plant height. To quantify plant height, the coleoptile was marked and plant height was measured from this mark to the tip of the longest leaf.

### Diversity of Root and Shoot Traits 3 Weeks after Emergence

In addition to axial and lateral root number determined from the μCT scans 3 weeks after emergence, final root branching density was determined by dividing lateral by axial root number. After the final CT scan, roots were gently washed out from the soil and dried at 60°C for at least 72 h before determining root dry weight. Shoot dry weight and root-shoot ratio were further plant vigor traits that were measured at the end of the experiment 3 weeks after emergence. As for roots, shoots were dried for 72 h before weighing. Four additional replications per bulk density of the variety “Arina” were grown under the exactly same conditions but were never scanned in order to check whether X-ray irradiation adversely affected plant development (Flavel et al., 2012). Comparing their root and shoot dry weights with plants from the same variety, which were regularly scanned, allowed excluding effects of X-ray on plant development (Supplementary Table 2). Final plant height, leaf and tiller number as well as root-shoot ratio were used as additional plant vigor indicators.

### Data Analysis and Statistics

Data analysis and statistics were performed in R version 3.1.3 (R Core Team, 2015). In order to account for effects of repeated measurements, root system and shoot development were evaluated in the ASReml package (Guilmour et al., 2009) for R with the following linear mixed effect model:

$$Y_{ijkl}=\mu +\alpha_{i}+\beta_{j}+{\alpha \beta }_{ij}+w_{l}+{\alpha \beta w}_{ijl}+p_{k}+\epsilon_{ijkl}$$

where *Y* represents the measured trait of the ith variety (*i* = 1,2,…,13, 14) and of the jth bulk density (*j* = 1.3 g cm^-3^, 1.45 g cm^-3^, 1.6 cm^-3^), within the 1th week after emergence (*l* = 1, 2, 3) and the kth pot (*k* = 1, 2,…, 167, 168); α is the variety effect, β is the effect of the soil bulk density, αβ is the interaction between the variety and the bulk density, *w* is the effect of the week after emergence, αβ*w* is the interaction between the variety, the bulk density and the week after emergence, β*w i*s the interaction between the bulk density and the week after emergence, *p* is the effect of the pot and ε is the residual error. Variety, bulk density, variety-bulk density interaction and the week after emergence were treated as fixed factors whereas the remaining factors were set as random. The numbers of axial and lateral root were converted by square root transformation to better meet the model (Eq. 1) assumptions (Supplementary Figure 1). Based on the model predictors for all variety-bulk density combinations, plant height and back-transformed root numbers were related using the following square root function:

$$h=a*r^{0.5}+b$$

where *h* is the plant height at 1, 2, or 3 weeks after emergence, *r* is the axial or lateral root number at 1, 2, or 3 weeks after emergence, *a* is the scaling factor and *b* the intercept. Performing analysis of covariance (ANCOVA) based on model predictors (Eq. 1) allowed determining whether bulk density significantly affected root-shoot relationships. To do so, soil bulk density and transformed (square root) root numbers were used as factorial and continuous variable, respectively and plant height was treated as response variable.

The set of root and shoot traits obtained 3 weeks after emergence was evaluated with a two-factorial analysis of variance (ANOVA), in which the effects of the variety, the soil bulk density and their interaction were treated as fixed effects. Again, the number of roots was converted by square root transformation (Supplementary Figure 2). To compare the relative genotypic variability of root numbers and root and shoot dry weight between different levels of soil strength, coefficients of variation (CV) were calculated for each treatment-level (*n* = 4). Using one factorial ANOVA, the relative genotypic variability of root and shoot traits could be compared across the three levels of soil strength studied. Means between soil bulk density levels and genotypes were compared using least significant difference (LSD) and Tukey’s honest significant difference (HSD) tests, respectively at significance level of *p* < 0.05. The tolerance of varieties to increased soil bulk density was assessed based on the proportion between variety mean trait values under high (1.6 g cm^-3^) or moderate (1.45 g cm^-3^) soil bulk density and low bulk density (1.3 g cm^-3^), respectively. In doing so, values were standardized in order to account for the effects of the breeding background (pre- and post-green revolution).

Furthermore, broad-sense heritability was calculated for root and shoot traits, which were quantified 3 weeks after emergence. To check for the stability of the inheritance under increased soil strength, heritability was calculated separately for each bulk density level. Genotypic variance was obtained by setting the replication and the variety as a fixed and random factor, respectively. Mean based heritability was estimated as proposed by Falconer and Mackay (1996):

$$H^{2}=\frac{\sigma_{g}^{2}}{\sigma_{g}^{2}+\frac{\sigma_{e}^{2}}{r}}$$

where σ_g_^2^ and σ_e_^2^ represents the genotype and residual error variance, respectively and *r* is the number of replications.

## Results

### Root System and Shoot Development in Response to Increased Soil Bulk Density

Soil bulk density significantly (*p* < 0.01) affected all root system and shoot traits, which were assessed in weekly intervals during plant growth (**Table 2**). Increased soil strength caused delayed plant development and resulted in decreased plant height, lower tiller, and leaf number as well as decreased axial and lateral root number. Compared to low and moderate soil bulk density, plant height and leaf number under high soil bulk density were decreased already 1 week after leaf emergence by 25–44%. Two and 3 weeks after emergence leaf number and plant height under high soil bulk density were around 35% lower compared to the plants grown under low and moderate bulk density (**Table 3**). Moderate soil compaction also led to a slight reduction in leaf number and plant height of around 7% compared to the low bulk density treatment. The strongest effects of increased soil strength on shoot development were observed for the number of tillers. Under low bulk density plants developed 2.7 and 4.0 tillers 2 and 3 weeks after emergence, respectively. These numbers decreased under moderate soil compaction to 2.0 and 3.0 tillers per plant 2 and 3 weeks after emergence, respectively, whereas under high soil bulk density almost no tillers were developed (**Table 3**). In terms of root number, the responses to increased soil bulk density were in a similar order of magnitude as for shoot traits. Under high soil strength, lateral root number decreased by around 70% compared to the plants grown at low bulk density at all three measurement points. A reduction of axial roots due to high soil bulk density could be observed 2 and 3 weeks after emergence (**Table 3**). Under moderate soil compaction axial root number decreased only slightly compared to the low bulk density treatment, whereas 2 and 3 weeks after emergence lateral root number was decreased by 11 and 19%, respectively (**Table 3**).

**Table 2:** Effects of variety (V), soil bulk density (BD), their interaction and week after emergence (WAE) on axial and lateral root number (NoAx and NoLat, respectively), plant height, number of fully developed leaves and number of tillers; significance of effects was determined using linear mixed model (Eq. 1) and Wald-tests; ^∗^ and ^∗∗^ denote significant effects at *p* < 0.05 and <0.01, respectively, n.s., denotes non-significant effects (*n* = 4).

Trait	Transformation	V	BD	V:BD	WAE
NoAx [#]	sqrt	^∗∗^	^∗^	n.s.	^∗∗^
NoLat [#]	sqrt	^∗∗^	^∗∗^	^∗∗^	^∗∗^
Plant height [mm]		^∗∗^	^∗∗^	^∗^	^∗∗^
Leaf number [#]		^∗∗^	^∗∗^	n.s.	^∗∗^
Tiller number [#]		^∗∗^	^∗∗^	^∗^	^∗^


**Table 3:** Bulk density mean values of predictors obtained from linear mixed model (Eq. 1) for axial and lateral root numbers (NoAx and NoLat, respectively), plant height, leaf and tiller number under low (1.3 g cm^-3^), moderate (1.45 g cm^-3^), and high (1.6 g cm^-3^) soil bulk density 1, 2, and 3 weeks after emergence (WAE); values are based on 4 replications of 14 varieties and root numbers represent back-transformed values.

	1 WAE			2 WAE			3 WAE		
			
Bulk density [g cm^-3^]	1.3	1.45	1.6	1.3	1.45	1.6	1.3	1.45	1.6
NoAx [#]	4.6	4.9	4.5	10.2	10.1	7.2	16.7	16.0	9.1
NoLat [#]	50.5	48.1	15.3	77.1	68.9	25.4	116.5	94.1	34.9
Plant height [mm]	187.1	176.5	132.4	282.0	261.4	168.4	320.1	292.9	181.4
Leaf number [#]	1.94	1.88	1.05	3.28	3.00	2.05	4.39	4.18	2.94
Tiller number [#]	0.36	0.34	0.00	2.70	2.05	0.02	4.03	3.07	0.06


**FIGURE 2:**
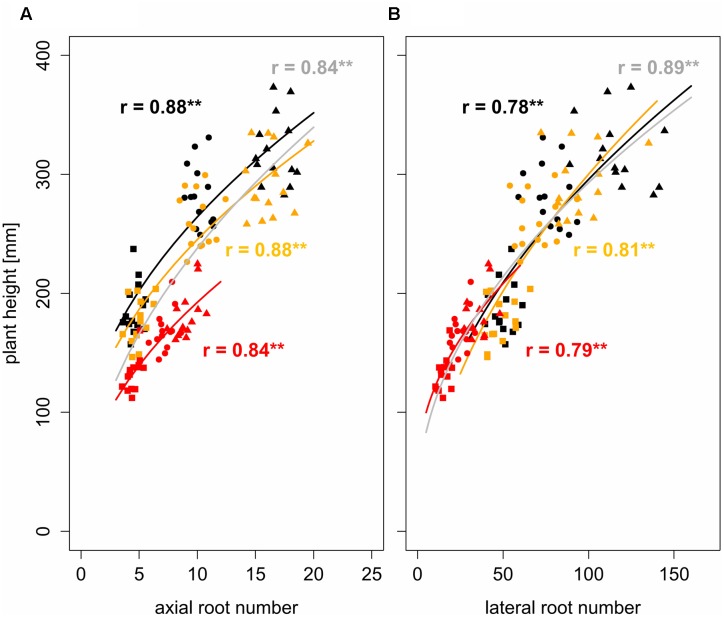
**Non-linear regressions and Pearson correlation coefficients between** **(A)** axial and **(B)** lateral root number and plant height under bulk densities of 1.3 g cm^-3^ (black), 1.45 g cm^-3^ (orange), and 1.6 g cm^-3^ (red), gray represents regression over all three bulk densities; square, circle, and triangle symbol shapes represent values obtained 1, 2, and 3 weeks after leaf emergence, respectively; ^∗∗^ denotes significant correlation at *p* < 0.01 (*n* = 4).

Using a square root function (Eq. 2) the development of lateral and axial root numbers was observed to be significantly (*p* < 0.01) correlated with the development of plant height. Pearson correlation coefficients were between 0.78 and 0.88 indicating a reasonably strong non-linear relationship between root number and plant height over time (**Figure 2**). Analysis of covariance with bulk density as a fixed factor allowed determining the influence of increased soil strength on root-shoot relationships. The relationship between the number of axial roots and plant height was affected significantly (*p* < 0.01) by bulk density but not by the interaction of bulk density and root number. Bulk density explained 16% of the total variance, whereas the interaction between bulk density and root number only explained 0.1% of the total variance (**Table 4**). In contrast to that, the relationship between the development of lateral root number and plant height remained unaffected by increased soil bulk density (**Table 4**). Hence, it can be concluded that more axial roots are needed to maintain shoot growth under increased soil strength compared to conditions of loose soil (**Figure 2**).

**Table 4:** Effects of soil bulk density, axial and lateral root numbers (NoAx and NoLat, respectively) based on linear mixed model predictors (Eq. 1) and their interaction on plant height using analysis of covariance model: bulk density (1.3, 1.45, and 1.6 g cm^-3^) was treated as a factor and transformed root number (square root) was treated as continuous variable; numbers indicate proportion of variance (SS_xx_/SS_tot_) in percentage explained by each effect; ^∗∗^ denotes significant effects at *p* < 0.01, n.s., denotes non-significant effects and *R*^2^ represents multiple *r*-squared.

Effect	SS_xx_/SS_tot_
Bulk density	16.3^∗∗^
NoAx^0.5^	70.6^∗∗^
Bulk density : NoAx^0.5^	0.1 n.s.
*R*^2^	0.87
Bulk density	0.5 n.s.
NoLat^0.5^	78.4^∗∗^
Bulk density : NoLat^0.5^	0.5 n.s.
*R*^2^	0.79


### Genotypic Diversity of Root and Shoot Traits under Different Levels of Soil Compaction

Besides increased soil strength, root system and shoot development were also influenced by the variety. Axial and lateral root number, plant height as well as leaf and tiller number were significantly (*p* < 0.01) different between the 14 investigated varieties (**Table 2**). As observed for the development of root systems and shoots, genetic diversity of root and shoot properties were also observed 3 weeks after emergence at the end of the experiment. Lateral and axial root number as well as the lateral–axial root number ratio was significantly different (*p* < 0.01) between the different varieties. The same responses were observed for shoot traits such as plant height, leaf and tiller number, root and shoot dry weight as well as root-shoot ratio (**Table 5**).

**Table 5:** Effects of variety (V), soil bulk density (BD) and their interaction on axial and lateral root number (NoAx and NoLat, respectively) and plant vigor parameters 3 weeks after emergence analyzed with analysis of variance (ANOVA); minimum (Min), average (Mean) and maximum (Max) values and Tukey honest significant difference (HSD) at *p* < 0.05, values in brackets for NoAx, NoLat, and NoLat/NoAx represent back-transformed values; ^∗^ and ^∗∗^ denote significant effects at *p* < 0.05 and <0.01 respectively, n.s., denotes non-significant effects (*n* = 4).

Trait	Transformation	V	BD	V:BD	Min	Mean	Max	HSD
NoAx [#]	sqrt	^∗∗^	^∗∗^	n.s.	2.69 (7.25)	3.70 (13.70)	4.49 (20.16)	0.68
NoLat [#]	sqrt	^∗∗^	^∗∗^	^∗∗^	5.27 (27.82)	8.80 (77.47)	12.29 (151.04)	1.57
NoLat/NoAx [#]	sqrt	^∗∗^	^∗∗^	^∗∗^	1.76 (3.10)	2.34 (5.48)	2.92 (8.54)	0.42
Root dry weight [g]		^∗∗^	^∗∗^	n.s	0.059	0.386	0.733	0.217
Shoot dry weight [g]		^∗∗^	^∗∗^	^∗^	0.097	0.454	0.767	0.148
Root-Shoot-Ratio [-]		^∗∗^	^∗∗^	n.s.	0.58	0.80	1.07	0.365
Plant height [mm]		^∗∗^	^∗∗^	^∗∗^	160.3	264.8	373.3	42.3
Leaf number [#]		^∗∗^	^∗∗^	^∗^	2.25	3.83	5.00	0.93
Tiller number [#]		^∗∗^	^∗∗^	^∗∗^	0.00	2.39	5.5	2.04


**FIGURE 3:**
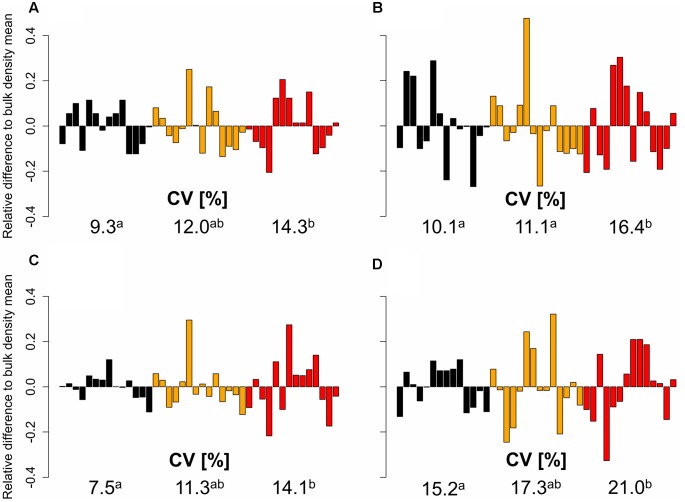
**Relative difference to the bulk density mean (black = 1.3 g cm^-3^, orange = 1.45 g cm^-3^, red = 1.6 g cm^-3^) of 14 genotypes for** **(A)** axial and **(B)** lateral root number, **(C)** shoot and **(D)** root dry weight based on genotype mean values (*n* = 4), represented by individual bars; coefficients of variation (CV) between different levels of soil bulk density were compared using least significant difference (LSD) tests at *p* < 0.05; significant differences are indicated with different letters.

Furthermore, the obtained data showed that the genotypic variability of root numbers and root and shoot dry weight increased with increasing soil strength. The relative genotypic difference of axial and lateral root number and root and shoot biomass to the respective bulk density mean value increased significantly with increasing bulk density (**Figure 3**). This effect of increasing genotypic variability with increasing soil strength was particularly pronounced for axial root number and root and shoot dry weight and less for lateral root number. Coefficients of variation for axial root number, which were calculated for each soil bulk density treatment were 9.3% under low soil strength and increased to 12.0 and 14.3% under moderate and high soil strength, respectively. Also for root and shoot dry weight, a significant increase of CV with increased soil compaction could be observed. Under low soil bulk density CV for root and shoot dry weight was 15.2 and 7.5% respectively. These values increased to 17.3 and 21% for root dry weight and 11.3 and 14.1% for shoot dry weight due to a moderate and severe increase in soil bulk density, respectively. For lateral root number bulk density mean CVs of 10.1, 11.1, and 16.4% were obtained under low, moderate, and high soil strength, respectively also indicating a slight increase of genotypic variability with increasing soil bulk density (**Figure 3**).

Estimations of broad-sense heritability (Eq. 3) showed that the degree of inheritance is comparable between shoot and root traits. Furthermore, it was observed that the heritability of most traits decreased only slightly in response to increased soil strength. Lateral root number and plant height showed heritabilities of more than 75% under all three soil compaction levels. For axial root number and the number of leaves, broad-sense heritability ranged from 58 to 75% and was also only slightly lower under high and moderate soil bulk density compared to low soil strength. The lowest heritability estimations were observed shoot dry weight under low soil bulk density (**Table 6**). Most likely this relatively low value of 48% was caused by the relatively low genotypic variance of shoot dry weight under low soil strength (**Figure 3**).

**Table 6:** Estimated broad sense heritability (Eq. 3) of axial and lateral root number (NoAx and NoLat, respectively) and plant vigor traits using variance components 3 weeks after shoot emergence; heritability based on means (*n* = 4) of 14 varieties was calculated separately for low (1.3 g cm^-3^), moderate (1.45 g cm^-3^), and high soil bulk density (1.6 g cm^-3^).

		Heritability		
		
Trait	Transformation	1.3 g cm^-3^	1.45 g cm^-3^	1.6 g cm^-3^
NoAx [#]	sqrt	0.67	0.66	0.56
NoLat [#]	sqrt	0.92	0.89	0.75
NoLat/NoAx [#]	sqrt	0.86	0.66	0.50
Root dry weight [g]		0.54	0.70	0.56
Shoot dry weight [g]		0.48	0.65	0.64
Plant height [mm]		0.95	0.91	0.93
Leaf number [#]		0.74	0.67	0.65


### Tolerance to Increased Soil Strength among Varieties

**FIGURE 4:**
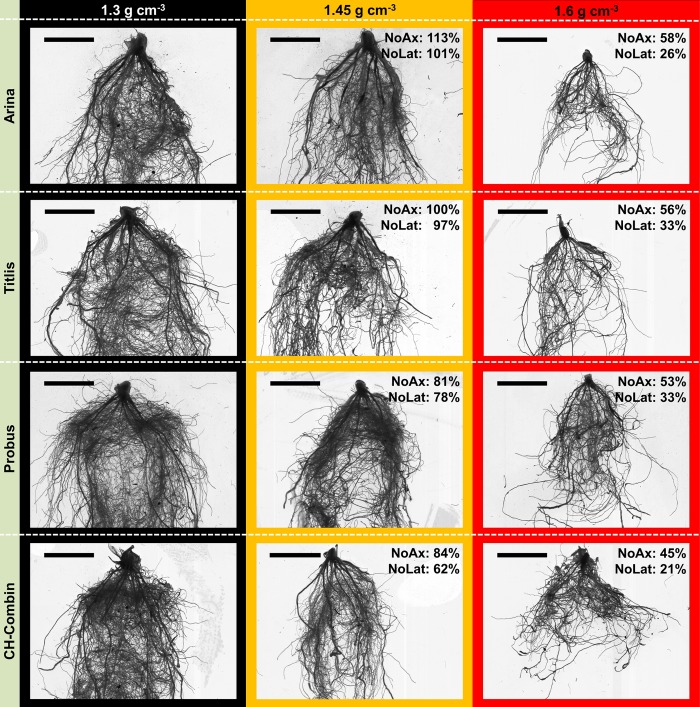
**Genetic diversity of root system phenotype among four selected genotypes under low (1.3 g cm^-3^), moderate (1.45 g cm^-3^) and high soil bulk density (1.6 g cm^-3^) 3 weeks after shoot emergence; “Arina” and “Titlis” represent cultivars, which are relatively tolerant to moderate soil compaction indicated by showing no or only small decrease in root numbers, “Probus” and “CH-Combin” represent cultivars, which are sensitive to moderate soil compaction; percentage values indicate the relative number of axial (NoAx) and lateral roots (NoLat) when comparing genotype mean values under moderate and high soil bulk density to low soil bulk density; scale bar = 3 cm**.

Besides significant genotypic responses to different levels of soil bulk density, root and shoot traits that were assessed 3 weeks after emergence were significantly affected by increased soil bulk density (**Table 5**). Furthermore, as illustrated in **Figure 4**, the magnitude of the responses in root numbers to soil compaction differed among the assessed varieties. Generally, increased soil strength resulted in decreased plant vigor and root numbers. Average shoot dry weight decreased by 19 and 82% under moderate and high soil strength, respectively when compared to plants grown under low bulk density. In comparison to plants from the low bulk density treatment, root dry weight was 36 and 87% lower due to moderate and high soil compaction, respectively (Supplementary Table 3). Therefore, root-shoot ratios decreased from 0.96 under low soil strength to 0.75 and 0.70 under moderate and high soil bulk density, respectively. Furthermore, lateral and axial root number as well as lateral–axial root number ratio decreased due to moderate and high soil compaction by 4–19% and 45–70%, respectively.

**FIGURE 5:**
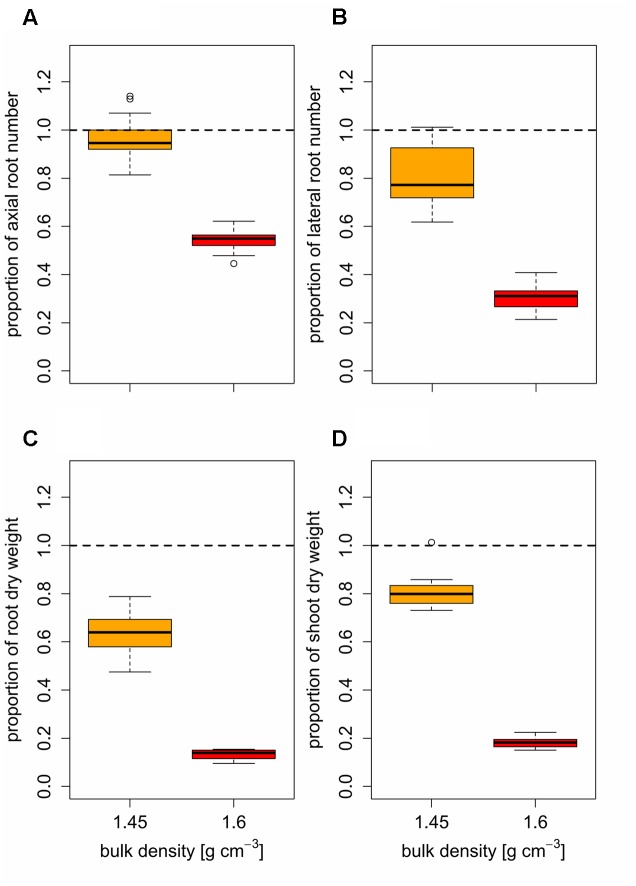
**Boxplots of relative** **(A)** axial and **(B)** lateral root numbers, **(C)** root and **(D)** shoot dry weight based on mean values (*n* = 4) of 14 genotypes under moderate (1.45 g cm^-3^, orange) and high bulk density (1.6 g cm^-3^, red), expressed as the ratio between genotype mean values under increased and low (1.3 g cm^-3^) bulk density; dashed line represents low bulk density.

Despite these general responses of root numbers and root and shoot dry weight to increased levels of soil compaction, the magnitude of these responses differed particularly under moderate soil compaction. The strongest responses to moderate soil compaction were observed in root dry weight, followed by lateral root number and shoot dry weight. The number of axial roots were least affected by the increase of soil bulk density from 1.3 to 1.45 g cm^-3^ (**Figure 5**). Furthermore, the effect of moderate soil compaction on axial and lateral root number varied considerably between different varieties. In certain varieties (Arina, Mont-Calme 268, Titlis) axial root number was not affected or even increased in response to moderately increased soil bulk density, whereas in other varieties (CH-Combin, Mont-Calme 245, Probus) the number of axial roots decreased by almost 20% (**Figure 5**). Similar responses were observed for the number of lateral roots, which decreased by more than 25% in some varieties (CH-Combin, Probus, Plantahof), whereas in other varieties (Arina, Mont-Calme 268, Titlis) the number of lateral roots remained constant under moderately increased soil strength (**Figure 5**). This genetic diversity in the responses to moderate soil compaction was also observed for shoot dry weight. The reduction of shoot dry weight due to moderately increased soil strength in the most sensitive varieties was more than 25%. In other varieties instead, moderately increased soil bulk density did not affect shoot dry weight (**Figure 5**). In contrast to root numbers and shoot dry weight, root dry weight was reduced in all varieties under moderate soil bulk density compared to the plants grown under low soil strength. However, also for responses of root dry weight to moderately increased bulk density considerable genetic diversity was observed (**Figure 5**). Under high soil strength root numbers and root and shoot dry weight decreased in all varieties compared to low and moderate soil compaction. As observed for plants grown under moderate soil strength, the responses of axial and lateral root number to high soil compaction among the investigated varieties covered a wider range than those of root and shoot dry weight (**Figure 5**).

**FIGURE 6:**
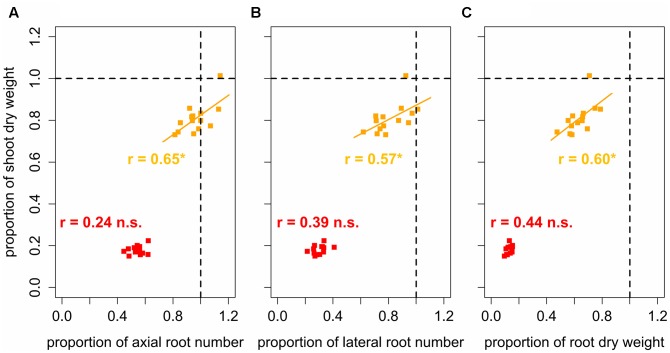
**Linear regressions and Pearson correlation coefficients between relative shoot dry weight and relative** **(A)** axial and **(B)** lateral root number and **(C)** relative root dry weight, expressed as the ratio between genotype mean values under increased (1.45 g cm^-3^, orange; 1.6 g cm^-3^, red) and low (1.3 g cm^-3^) bulk density represented by dashed lines; ^∗^ denotes significant correlation at *p* < 0.05, n.s., denotes non-significant correlations (*n* = 4).

**FIGURE 7:**
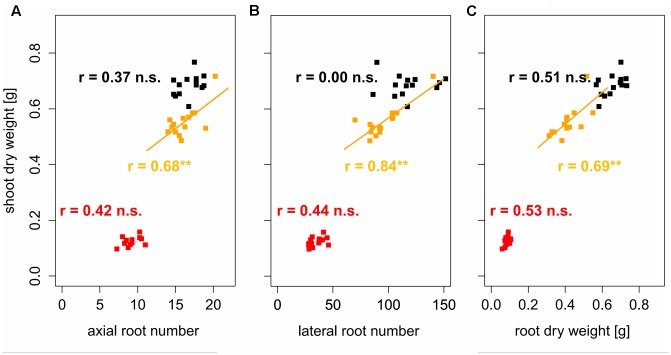
**Linear regressions and Pearson correlation coefficients between shoot dry weight and** **(A)** axial and **(B)** lateral root number and **(C)** root dry weight; black, orange, and red symbols represent soil bulk density of 1.3, 1.45, and 1.6 g cm^-3^, respectively, ^∗∗^ denotes significant correlation at *p* < 0.01, n.s., denotes non-significant correlations (*n* = 4).

To assess the tolerance of the different varieties to soil compaction, correlations between root traits and shoot dry weight were performed based on proportions of the respective traits. This was achieved by dividing the variety mean value obtained under moderate or high soil bulk density by the variety mean value under low soil bulk density. In doing so, values could be standardized and corrected for the influence of the different breeding background. These analyses showed significant (*p* < 0.05) positive correlations between the root system traits and shoot dry weight under moderate soil bulk density. Proportions of shoot dry weight, were positively correlated (0.57 < *r* < 0.65) with proportions of axial and lateral root number as well as with proportions of root dry weight (**Figure 6**). Shoot dry weight of varieties, which maintained or increased their axial root number under moderate soil compaction, decreased only slightly even though the respective root dry weight decreased by more than 20%. The same but less pronounced findings were obtained for the relationship between relative lateral root number and relative shoot dry weight under moderately increased soil bulk density. Under high soil strength, no significant correlation between proportions of shoot dry weight and root numbers were found (**Figure 6**). The same results were achieved, when correlating actual root numbers and root dry weight to actual shoot dry weight. Under moderate soil compaction increased numbers of roots as well as root dry weight were related to increased shoot dry weight (0.68 < *r* < 0.84). Yet under high and low soil bulk density no significant relationship between below and above ground traits was (**Figure 7**). These results showed that the tolerance to soil compaction differs significantly among wheat varieties and that root numbers are suitable to explain this genetic diversity.

## Discussion

The main aim of the current study was to evaluate the suitability of root number as a target trait for improving the tolerance to soil compaction. Quantifying root system and shoot development across 14 wheat varieties grown under three levels of soil bulk density revealed how root-shoot relationships are affected by increasing soil strength. Furthermore, it was possible to show that root numbers are heritable and may explain the genotypic tolerance to compacted soils.

### Axial Root Number Determines Shoot Growth Dynamics under Increased Soil Bulk Density

To better understand how increased soil strength affects whole plant growth and root-shoot relationships, lateral and axial root number as well as plant height, leaf and tiller number were quantified in weekly intervals. Compared to the columns packed to 1.3 g cm^-3^, mechanical impedance increased by around 30 and 200% under moderate and high soil bulk density, respectively. This increase of soil strength due to higher soil bulk density is comparable to previous studies (Grzesiak et al., 2014; Nosalewicz and Lipiec, 2014; Colombi and Walter, 2016) and significantly affected root system and shoot development (**Table 2**). Shoot development was delayed due to increased soil strength (**Table 3**), which corresponds to previous studies in wheat, barley, and maize (Masle and Passioura, 1987; Beemster et al., 1996; Young et al., 1997). Similar to shoot growth, root system development was delayed due to increased soil bulk density. As observed previously in different small grain cereals (Colombi and Walter, 2016) the initiation of axial and lateral roots was deferred in response to increased soil strength at early stages of plant development (**Table 3**).

The simultaneous quantification of plant height and root numbers in response to increased soil bulk density over time enabled to relate above and below ground growth. These results showed, that under all levels of soil strength, axial and lateral root numbers were highly correlated (0.78 < *r* < 0.88) with plant height following a square root function (**Figure 2**). Similar results were obtained in broccoli seedlings, where it has been shown that root length explained responses of leaf area to soil compaction (Montagu et al., 2001). In this and other studies (Grzesiak et al., 2014; Nosalewicz and Lipiec, 2014) root traits were not assessed continuously during growth, which does not allow investigating how soil strength affects the dynamics of root-shoot relationship. If soil bulk density increased, more axial roots were needed to maintain shoot growth (**Figure 2**), whereas no such effect could be observed for the relationship between lateral root number and plant height (**Table 4**). This finding demonstrated that early plant development under increased soil strength was mainly driven by axial and only partially by lateral roots. Most probably this was caused by the fact that thick roots are less prone to buckling compared to thin roots (Materechera et al., 1992; Lipiec et al., 2012; Chimungu et al., 2015). Hence, the obtained results indicate that axial roots, which are inherently thicker than lateral roots, were of increasing importance for early plant vigor when soil strength increases.

### Root Numbers Are Suited to Assess Tolerance to Increased Soil Bulk Density

Root system and shoot development (**Table 2**) as well as root and shoot traits obtained 3 weeks after emergence (**Table 5**) were significantly different among the 14 investigated varieties. Genotypic differences in response to increased soil strength were observed in maize, wheat, and triticale (Bushamuka and Zobel, 1998; Kubo et al., 2004, 2006; Grzesiak et al., 2014), whereas other studies did not report significant differences between genotypes (Tracy et al., 2012). Certain studies investigated only two to four genotypes (Bushamuka and Zobel, 1998; Tracy et al., 2012; Grzesiak et al., 2014), which did not allow relating root traits to genotypic plant performance or compaction was simulated using paraffin-Vaseline disks instead of using actually compacted soil (Kubo et al., 2004, 2006). The results obtained in the current study revealed a genetic diversity of root and shoot traits in wheat in response to soil compaction. Besides this genetic diversity of root and shoot traits (**Table 5**), increasing variability of traits among varieties were observed due to increased soil bulk density, what corresponds to previous studies (Bushamuka and Zobel, 1998; Grzesiak et al., 2014). Compared to loosely packed soil, the genetic variability of axial and lateral root number 3 weeks after emergence increased under moderate and high soil bulk density, which is indicated by the increase of coefficients of variation with increasing soil strength (**Figure 3**). Also genotypic diversity of root and shoot dry weight at the end of the experiment was higher under moderate and high soil strength compared to plants grown under low soil bulk density (**Figure 3**).

Below and above ground responses to compacted soil were observed to differ between varieties, as illustrated in **Figure 4** and the magnitude of these responses was different for root numbers and shoot dry weight when compared to root dry weight (**Figure 5**). Under moderate soil compaction some varieties increased their root numbers and shoot dry weight remained unaffected. In other varieties instead, already a moderate increase of soil strength led to a reduction of root numbers and shoot dry weight of 18% to more than 30% (**Figure 5**). Decreasing, constant or even increasing plant vigor in response to moderate soil compaction was observed in wheat, barley, and maize (Bushamuka and Zobel, 1998; Bingham and Bengough, 2003; Grzesiak et al., 2014; Nosalewicz and Lipiec, 2014). Relative root dry weight instead was reduced in all varieties due to moderately increased soil compaction (**Figure 5**), resulting in reduced root-shoot ratio under moderate compared to low soil bulk density. The discrepancy between root numbers and root dry weight in response to moderate soil compaction can be most likely explained by decreased root length, which was observed in response to compaction in a wide range of monocot crop species (Bingham and Bengough, 2003; Tracy et al., 2012; Grzesiak et al., 2014). Under high soil bulk density similar but more severe responses were observed. Compared to low and moderate soil bulk density, root and shoot biomass as well as root numbers decreased in all genotypes and the strongest response was observed for root dry weight followed by shoot biomass and root numbers (**Figure 5**).

Previous studies observed that root numbers influence crop performance under abiotic stress including low water availability (Zhan et al., 2015; Gao and Lynch, 2016) or low levels of plant available soil nitrogen (Saengwilai et al., 2014; Zhan and Lynch, 2015) and phosphorus (Miguel et al., 2013). Furthermore, the assessment of root numbers is possible under field conditions at high throughputs in a wide range of mono- and dicotyledonous crops (Trachsel et al., 2011; Colombi et al., 2015; Burridge et al., 2016; Colombi and Walter, 2016). Measurements of root biomass or root length in the field instead are challenging and laborious. Results from the current study showed that axial and lateral root numbers not only respond to soil compaction but also determine early shoot vigor under moderately increased soil strength. Similar to results reported for narrow-leafed lupins (Chen Y.L. et al., 2014), variety mean values of relative axial and lateral root number were significantly related (0.57 < *r* < 0.65, *p* < 0.05) to relative shoot dry weight (**Figure 6**). Varieties, which maintained their root numbers under moderate soil compaction compared to conditions of loose soil showed less decreasing shoot dry weight than varieties, in which moderate compaction led to a decrease of root numbers. Remarkably, this relationship could be shown even though root dry weight decreased in all assessed varieties due to moderate soil compaction (**Figure 6**). Also absolute root numbers were positively correlated to shoot dry weight (0.68 < *r* < 0.84, *p* < 0.01) under moderately increased soil strength (**Figure 7**). Under severe soil compaction no significant relationship between shoot dry weight and root numbers or root dry weight could be observed (**Figures 6**, **7**). Mechanical impedance in the severely compacted columns was most likely above the limit at which genotypic differences affect shoot performance (Kubo et al., 2004). Furthermore, calculations of broad-sense heritability showed that root numbers are supposed to be relatively highly heritable. Even under high soil strength, estimations of 56 and 75% heritability were obtained for axial and lateral root number, which was in a similar range than the values observed for plant height, leaf number or shoot dry weight (**Table 6**). These results are comparable to other studies, in which heritability estimations for shoot and root traits were evaluated in mono- and dicotyledonous crops (Wilcox and Farmer, 1968; Bucksch et al., 2014; Colombi et al., 2015; Li et al., 2015; Richard et al., 2015; Burridge et al., 2016). Taking into account these heritability estimations and the significant influence of root number on shoot biomass, the number of roots is suggested to be a suitable indicator to assess the genotypic tolerance to soil compaction.

## Conclusion

With the current study we demonstrated that early shoot development of wheat on compacted soil is closely related to the number of axial roots. Thereby significant genetic variability between 14 different wheat varieties could be shown with respect to their tolerance to increased soil bulk density. In particular under moderate soil compaction, the genotypic capacity to maintain the number of axial and lateral roots resulted in higher shoot biomass production. Furthermore, root numbers showed relatively high heritabilities and can be assessed at high throughput rates even under field conditions. Therefore, it can be stated that root number is a promising target trait for crop breeding programs aiming to improve the tolerance of crops to compacted soil. For future studies, it would be desirable to evaluate whether the presented findings are transferable to other species and to quantify root-soil interactions underlying crop productivity on compacted soils.

## Author Contributions

TC and AW designed the study, TC performed experiments and data analysis, TC and AW interpreted the data and wrote the manuscript. All authors approved the final version.

## Conflict of Interest Statement

The authors declare that the research was conducted in the absence of any commercial or financial relationships that could be construed as a potential conflict of interest.

## References

[B1] Abbott L. K. Murphy D. V. (2007). *Soil Biological Fertility. A Key to Sustainable Land Use in Agriculture.* Berlin: Springer.

[B2] Arvidsson J. Etana A. Rydberg T. (2014). Crop yield in Swedish experiments with shallow tillage and no-tillage 1983–2012. *Eur. J. Agron.* 52 307–315. 10.1016/j.eja.2013.08.002

[B3] Arvidsson J. Håkansson I. (2014). Response of different crops to soil compaction—Short-term effects in Swedish field experiments. *Soil Tillage Res.* 138 56–63. 10.1016/j.still.2013.12.006

[B4] Barraclough P. B. Weir A. H. (1988). Effects of a compacted subsoil layer on root and shoot growth, water use and nutrient uptake of winter wheat. *J. Agric. Sci.* 110 207–216. 10.1017/S0021859600081235

[B5] Batey T. (2009). Soil compaction and soil management - a review. *Soil Use Manag.* 25 335–345. 10.1111/j.1475-2743.2009.00236.x

[B6] Beemster G. T. S. Masle J. Williamson R. E. Farquhar G. D. (1996). Effects of soil resistance to root penetration on leaf expansion in wheat (*Triticum aestivum* L): kinematic analysis of leaf elongation. *J. Exp. Bot.* 47 1663–1678. 10.1093/jxb/47.11.1663

[B7] Bengough A. G. McKenzie B. M. Hallett P. D. Valentine T. A. (2011). Root elongation, water stress, and mechanical impedance: a review of limiting stresses and beneficial root tip traits. *J. Exp. Bot.* 62 59–68. 10.1093/jxb/erq350 21118824

[B8] Bengough A. G. Mullins C. E. (1991). Penetrometer resistance, root penetration resistance and root elongation rate in two sandy loam soils. *Plant Soil* 131 59–66. 10.1007/BF00010420

[B9] Bingham I. J. Bengough A. G. (2003). Morphological plasticity of wheat and barley roots in response to spatial variation in soil strength. *Plant Soil* 250 273–282. 10.1023/A:1022891519039

[B10] Bishopp A. Lynch J. P. (2015). The hidden half of crop yields. *Nat. Plants* 1:15117 10.1038/nplants.2015.117 27250548

[B11] Botta G. F. Tolon-Becerra A. Lastra-Bravo X. Tourn M. (2010). Tillage and traffic effects (planters and tractors) on soil compaction and soybean (*Glycine max* L.) yields in Argentinean pampas. *Soil Tillage Res.* 110 167–174. 10.1016/j.still.2010.07.001

[B12] Bottinelli N. Hallaire V. Goutal N. Bonnaud P. Ranger J. (2014). Impact of heavy traffic on soil macroporosity of two silty forest soils: Initial effect and short-term recovery. *Geoderma* 21 10–17. 10.1016/j.geoderma.2013.10.025

[B13] Bucksch A. Burridge J. York L. M. Das A. Nord E. Weitz J. S. (2014). Image-based high-throughput field phenotyping of crop roots. *Plant Physiol.* 166 470–486. 10.1104/pp.114.243519 25187526 PMC4213080

[B14] Burridge J. Jochua C. N. Bucksch A. Lynch J. P. (2016). Legume shovelomics: high-throughput phenotyping of common bean (*Phaseolus vulgaris* L.) and cowpea (*Vigna unguiculata* subsp, *unguiculata*) root architecture in the field. *Field Crops Res.* 192 21–32. 10.1016/j.fcr.2016.04.008

[B15] Bushamuka V. N. Zobel R. W. (1998). Differential genotypic and root type penetration of compacted soil layers. *Crop Sci.* 38 776–781. 10.2135/cropsci1998.0011183X003800030026x

[B16] Chen G. Weil R. R. Hill R. L. (2014). Effects of compaction and cover crops on soil least limiting water range and air permeability. *Soil Tillage Res.* 136 61–69. 10.1016/j.still.2013.09.004

[B17] Chen Y. L. Palta J. Clements J. Buirchell B. Siddique K. H. M. Rengel Z. (2014). Root architecture alteration of narrow-leafed lupin and wheat in response to soil compaction. *Field Crops Res.* 165 61–70. 10.1016/j.fcr.2014.04.007

[B18] Chimungu J. G. Loades K. W. Lynch J. P. (2015). Root anatomical phenes predict root penetration ability and biomechanical properties in maize (*Zea Mays*). *J. Exp. Bot.* 66 3151–3162. 10.1093/jxb/erv121 25903914 PMC4449537

[B19] Colombi T. Braun S. Keller T. Walter A. (2017). Artificial macropores attract crop roots and enhance plant productivity on compacted soils. *Sci. Total Environ.* 574 1283–1293. 10.1016/j.scitotenv.2016.07.194 27712865

[B20] Colombi T. Kirchgessner N. Le Marié C. A. York L. M. Lynch J. P. Hund A. (2015). Next generation shovelomics: set up a tent and REST. *Plant Soil* 388 1–20. 10.1007/s11104-015-2379-7

[B21] Colombi T. Walter A. (2016). Root responses of triticale and soybean to soil compaction in the field are reproducible under controlled conditions. *Funct. Plant Biol.* 43 114–128. 10.1071/FP15194 32480446

[B22] Falconer D. S. Mackay T. F. (1996). *Introduction to Quantitative Genetics*, 4th Edn Harlow: Longman.

[B23] Flavel R. Guppy C. Tighe M. (2012). Non-destructive quantification of cereal roots in soil using high-resolution X-ray tomography. *J. Exp. Bot.* 63 2503–2511. 10.1093/jxb/err421 22271595

[B24] Gao Y. Lynch J. P. (2016). Reduced crown root number improves water acquisition under water deficit stress in maize (*Zea mays* L.). *J. Exp. Bot.* 67 4545–4557. 10.1093/jxb/erw243 27401910 PMC4973737

[B25] Grzesiak M. T. Ostrowska A. Hura K. Rut G. Janowiak F. Rzepka A. (2014). Interspecific differences in root architecture among maize and triticale genotypes grown under drought, waterlogging and soil compaction. *Acta Physiol. Plant.* 36 3249–3261. 10.1007/s11738-014-1691-9

[B26] Guilmour A. R. Gogel B. J. Cullis B. R. Thompson R. (2009). *ASReml User Guide Release 3.0.* Hemel Hempstead: VSN International Ltd.

[B27] Hamza M. A. Anderson W. K. (2005). Soil compaction in cropping systems. *Soil Tillage Res.* 82 121–145. 10.1016/j.still.2004.08.009

[B28] Jähne B. (2002). *Digital Image Processing 5th Revised and Extended Edition*, 5th Edn Berlin: Springer-Verlag, 10.1007/3-540-27563-0

[B29] Jin K. Shen J. Ashton R. W. Dodd I. C. Parry M. A. J. Whalley W. R. (2013). How do roots elongate in a structured soil? *J. Exp. Bot.* 64 4761–4777. 10.1093/jxb/ert286 24043852

[B30] Kubo K. Iwama K. Yanagisawa A. Watanabe Y. Terauchi T. Jitsuyama Y. (2006). Genotypic variation of the ability of root to penetrate hard soil layers among Japanese wheat cultivars. *Plant Prod. Sci.* 9 47–55. 10.1626/Pps.9.47

[B31] Kubo K. Jitsuyama Y. Iwama K. Hasegawa T. Watanabe N. (2004). Genotypic difference in root penetration ability by durum wheat (*Triticum turgidum* L. var. durum) evaluated by a pot with paraffin-Vaseline discs. *Plant Soil* 262 169–177. 10.1023/B:PLSO.0000037033.23964.54

[B32] Kuncoro P. H. Koga K. Satta N. Muto Y. (2014a). A study on the effect of compaction on transport properties of soil gas and water I: relative gas diffusivity, air permeability, and saturated hydraulic conductivity. *Soil Tillage Res.* 143 172–179. 10.1016/j.still.2014.02.006

[B33] Kuncoro P. H. Koga K. Satta N. Muto Y. (2014b). A study on the effect of compaction on transport properties of soil gas and water. II: soil pore structure indices. *Soil Tillage Res.* 143 180–187. 10.1016/j.still.2014.01.008

[B34] Li R. Zeng Y. Xu J. Wang Q. Wu F. Cao M. (2015). Genetic variation for maize root architecture in response to drought stress at the seedling stage. *Breed. Sci.* 65 298–307. 10.1270/jsbbs.65.298 26366112 PMC4542930

[B35] Lipiec J. Hatano R. (2003). Quantification of compaction effects on soil physical properties and crop growth. *Geoderma* 116 107–136. 10.1016/S0016-7061(03)00097-1

[B36] Lipiec J. Horn R. Pietrusiewicz J. Siczek A. (2012). Effects of soil compaction on root elongation and anatomy of different cereal plant species. *Soil Tillage Res.* 121 74–81. 10.1016/j.still.2012.01.013

[B37] Lynch J. P. (1995). Root architecture and plant productivity. *Plant Physiol.* 109 7–13. 10.1104/pp.109.1.7 12228579 PMC157559

[B38] Masle J. Passioura J. (1987). The effect of soil strength on the growth of young wheat plants. *Aust. J. Plant Physiol.* 14 643–656. 10.1071/PP9870643

[B39] Materechera S. A. Alston A. M. Kirby J. M. Dexter A. R. (1992). Influence of root diameter on the penetration of seminal roots into a compacted subsoil. *Plant Soil* 144 297–303. 10.1007/BF00012888

[B40] Miguel M. A. Widrig A. Vieira R. F. Brown K. M. Lynch J. P. (2013). Basal root whorl number: a modulator of phosphorus acquisition in common bean (*Phaseolus vulgaris*). *Ann. Bot.* 112 973–982. 10.1093/aob/mct164 23925972 PMC3783229

[B41] Montagu K. D. Conroy J. P. Atwell B. J. (2001). The position of localized soil compaction determines root and subsequent shoot growth responses. *J. Exp. Bot.* 52 2127–2133. 10.1093/jexbot/52.364.2127 11604451

[B42] Nosalewicz A. Lipiec J. (2014). The effect of compacted soil layers on vertical root distribution and water uptake by wheat. *Plant Soil* 375 229–240. 10.1007/s11104-013-1961-0

[B43] Pfeifer J. Faget M. Walter A. Blossfeld S. Fiorani F. Schurr U. (2014). Spring barley shows dynamic compensatory root and shoot growth responses when exposed to localised soil compaction and fertilisation. *Funct. Plant Biol.* 41 581–597. 10.1071/FP13224 32481015

[B44] R Core Team (2015). *R: A Language and Environment for Statistical Computing.* Available at: http://www.r-project.org

[B45] Richard C. A. Hickey L. T. Fletcher S. Jennings R. Chenu K. Christopher J. T. (2015). High-throughput phenotyping of seminal root traits in wheat. *Plant Methods* 11:13 10.1186/s13007-015-0055-9 PMC4351910 25750658

[B46] Saengwilai P. Tian X. Lynch J. P. (2014). Low crown root number enhances nitrogen acquisition from low-nitrogen soils in maize. *Plant Physiol.* 166 581–589. 10.1104/pp.113.232603 24706553 PMC4213090

[B47] Schjonning P. Rasmussen K. J. (2000). Soil strength and soil pore characteristics for direct drilled and ploughed soils. *Soil Tillage Res.* 57 69–82. 10.1016/S0167-1987(00)00149-5

[B48] Trachsel S. Kaeppler S. M. Brown K. M. Lynch J. P. (2011). Shovelomics: high throughput phenotyping of maize (*Zea mays* L.) root architecture in the field. *Plant Soil* 341 75–87. 10.1007/s11104-010-0623-8

[B49] Tracy S. R. Black C. R. Roberts J. A. McNeill A. Davidson R. Tester M. (2012). Quantifying the effect of soil compaction on three varieties of wheat (*Triticum aestivum* L.) using X-ray Micro Computed Tomography (CT). *Plant Soil* 353 195–208. 10.1007/s11104-011-1022-5

[B50] Tracy S. R. Black C. R. Roberts J. A. Mooney S. J. (2011). Soil compaction: a review of past and present techniques for investigating effects on root growth. *J. Sci. Food Agric.* 91 1528–1537. 10.1002/jsfa.4424 21538366

[B51] Tracy S. R. Black C. R. Roberts J. A. Mooney S. J. (2013). Exploring the interacting effect of soil texture and bulk density on root system development in tomato (*Solanum lycopersicum* L.). *Environ. Exp. Bot.* 91 38–47. 10.1016/j.envexpbot.2013.03.003

[B52] Walter A. Liebisch F. Hund A. (2015). Plant phenotyping: from bean weighing to image analysis. *Plant Methods* 11:14 10.1186/s13007-015-0056-8 PMC4357161 25767559

[B53] Wilcox J. R. Farmer R. E. (1968). Heritability and C effects in early root growth of eastern cottonwood cuttings. *Heredity* 23 239–245. 10.1038/hdy.1968.31

[B54] York L. M. Nord E. A. Lynch J. P. (2013). Integration of root phenes for soil resource acquisition. *Front. Plant Sci.* 4:355 10.3389/fpls.2013.00355 PMC3771073 24062755

[B55] Young I. M. Montagu K. Conroy J. Bengough A. G. (1997). Mechanical impedance of root growth directly reduces leaf elongation rates of cereals. *New Phytol.* 135 613–619. 10.1046/j.1469-8137.1997.00693.x

[B56] Zhan A. Lynch J. P. (2015). Reduced frequency of lateral root branching improves N capture from low-N soils in maize. *J. Exp. Bot.* 66 2055–2065. 10.1093/jxb/erv007 25680794 PMC4378636

[B57] Zhan A. Schneider H. Lynch J. P. (2015). Reduced lateral root branching density improves drought tolerance in maize. *Plant Physiol.* 168 1603–1615. 10.1104/pp.15.00187 26077764 PMC4528736

