# Supplementary material for: Genetic Diversity under Soil Compaction in Wheat: Root Number as a Promising Trait for Early Plant Vigor
Source: Front Plant Sci. 2017 Mar 28;8:420. doi: 10.3389/fpls.2017.00420 (PMC5368237; doi:10.3389/fpls.2017.00420)
Supplement: Supplementary file 1 [file Table_1.DOC]

Supplementary Table 1: Settings of X-ray computed tomography scanner and reconstruction details.

| **Parameter** | **Setting** |
| --- | --- |
| Images scan [#] | 1600 |
| Averaged images [#] | 1 |
| Skipped images [#] | 0 |
| Current [μA] | 450 |
| Voltage [kV] | 120 |
| Illumination time per image [ms] | 131 |
| Filter | 0.1 mm copper |
| Binning 2 by 2 voxel | yes |
| Scanning time [min] | 7 |
| Voxel edge length [μm] | 68 |
| Beam hardening corrections | 3.6 |
