# Supplementary material for: Genetic Diversity under Soil Compaction in Wheat: Root Number as a Promising Trait for Early Plant Vigor
Source: Front Plant Sci. 2017 Mar 28;8:420. doi: 10.3389/fpls.2017.00420 (PMC5368237; doi:10.3389/fpls.2017.00420)
Supplement: Supplementary file 2 [file Table_2.DOC]

Supplementary Table 2: Effects of radiation exposure (X-ray), soil bulk density (BD) and their interaction in the variety “Arina” on root and shoot dry weight; ** denotes significant differences at p-level<0.01, n.s. denotes non-significant responses (n=4).

| **Trait** | **X-Ray** | **BD** | **X-ray:BD** |
| --- | --- | --- | --- |
| Root dry weight [g] | n.s. | ** | n.s. |
| Shoot dry weight [g] | n.s. | ** | n.s. |
