# Supplementary material for: Genetic Diversity under Soil Compaction in Wheat: Root Number as a Promising Trait for Early Plant Vigor
Source: Front Plant Sci. 2017 Mar 28;8:420. doi: 10.3389/fpls.2017.00420 (PMC5368237; doi:10.3389/fpls.2017.00420)
Supplement: Supplementary file 3 [file Table_3.DOC]

Supplementary Table 3: Summary of variety mean values (n=4) of axial and lateral root numbers (NoAx and NoLat, respectively), root and shoot dry weight (RootDW and ShootDW, respectively) under low (1.3 g cm^-3^), moderate (1.45 g cm^-3^) and high (1.6 g cm^-3^) soil bulk density three weeks after emergence and bulk density mean values (Average).

|  | **NoAx [#]** | | | **NoLat [#]** | | | **RootDW [g]** | | | **ShootDW [g]** | | |
| --- | --- | --- | --- | --- | --- | --- | --- | --- | --- | --- | --- | --- |
| **Variety** | **1.3** | **1.45** | **1.6** | **1.3** | **1.45** | **1.6** | **1.3** | **1.45** | **1.6** | **1.3** | **1.45** | **1.6** |
| Arina | 15.5 | 17.5 | 9.0 | 106.3 | 107.5 | 28.0 | 0.568 | 0.448 | 0.078 | 0.686 | 0.586 | 0.113 |
| CHClaro | 17.8 | 16.8 | 8.5 | 146.0 | 103.5 | 38.0 | 0.697 | 0.410 | 0.074 | 0.695 | 0.570 | 0.128 |
| CHCombin | 18.5 | 15.5 | 8.3 | 143.5 | 88.8 | 30.8 | 0.661 | 0.314 | 0.100 | 0.677 | 0.504 | 0.117 |
| Forel | 15.0 | 15.0 | 7.3 | 105.8 | 92.3 | 28.5 | 0.613 | 0.340 | 0.059 | 0.646 | 0.516 | 0.097 |
| Mont-Calme 245 | 18.8 | 16.0 | 10.3 | 109.8 | 103.8 | 44.8 | 0.653 | 0.407 | 0.079 | 0.718 | 0.566 | 0.138 |
| Mont-Calme 268 | 17.8 | 20.3 | 11.0 | 151.5 | 140.3 | 46.0 | 0.729 | 0.517 | 0.081 | 0.708 | 0.717 | 0.112 |
| Plantahof | 16.5 | 16.3 | 10.3 | 124.0 | 91.8 | 41.5 | 0.701 | 0.486 | 0.092 | 0.705 | 0.535 | 0.158 |
| Probus | 17.5 | 14.3 | 9.3 | 89.5 | 69.8 | 29.8 | 0.701 | 0.409 | 0.105 | 0.767 | 0.561 | 0.131 |
| Runal | 17.8 | 19.0 | 9.3 | 121.5 | 93.0 | 40.5 | 0.706 | 0.409 | 0.105 | 0.685 | 0.530 | 0.130 |
| Simano | 18.8 | 17.3 | 10.5 | 116.0 | 103.5 | 37.5 | 0.733 | 0.549 | 0.103 | 0.683 | 0.586 | 0.134 |
| Suretta | 14.8 | 14.0 | 8.0 | 117.3 | 84.3 | 31.3 | 0.579 | 0.329 | 0.089 | 0.703 | 0.517 | 0.141 |
| Titlis | 14.8 | 14.8 | 8.3 | 86.0 | 83.5 | 28.5 | 0.595 | 0.395 | 0.088 | 0.652 | 0.544 | 0.117 |
| Zenith | 15.5 | 14.5 | 8.8 | 112.5 | 85.5 | 31.8 | 0.643 | 0.424 | 0.074 | 0.654 | 0.534 | 0.103 |
| Zinal | 16.8 | 15.8 | 9.3 | 117.0 | 83.3 | 37.3 | 0.582 | 0.382 | 0.090 | 0.609 | 0.486 | 0.119 |
| **Average** | 16.9 | 16.2 | 9.2 | 117.6 | 95.1 | 35.3 | 0.654 | 0.416 | 0.087 | 0.685 | 0.554 | 0.124 |
