# Supplementary material for: Genetic Diversity under Soil Compaction in Wheat: Root Number as a Promising Trait for Early Plant Vigor
Source: Front Plant Sci. 2017 Mar 28;8:420. doi: 10.3389/fpls.2017.00420 (PMC5368237; doi:10.3389/fpls.2017.00420)
Supplement: Supplementary file 4 [file Image_1.PDF]

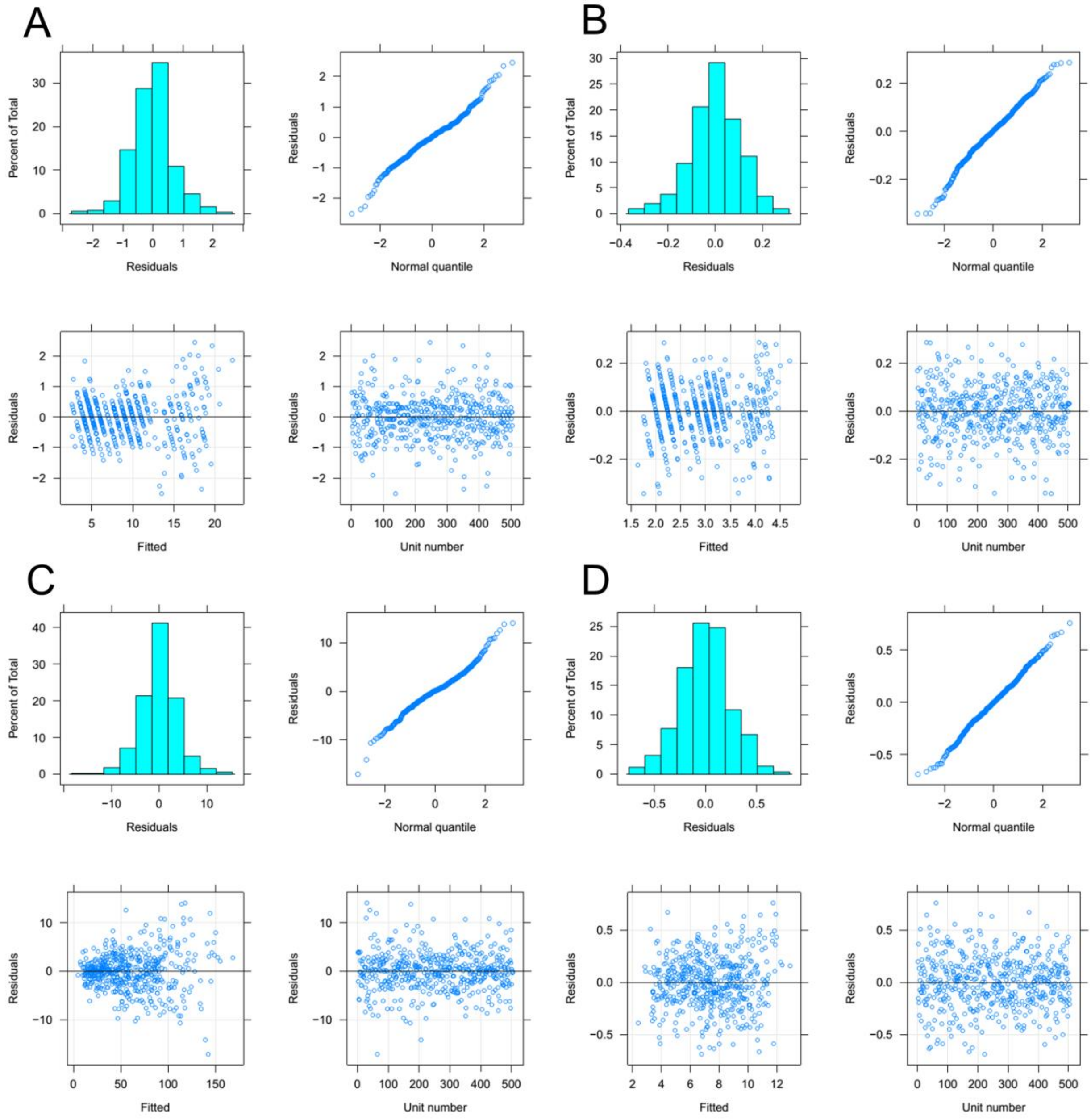

Supplementary Figure 1: Residual plots from linear mixed models (Eqn 1) using ASReml for R for (A) and (B) axial root number and (C) and (D) lateral root number (A) and (C) before square root transformation and (B) and (D) after square root transformation (n=4).
