# Supplementary material for: Genetic Diversity under Soil Compaction in Wheat: Root Number as a Promising Trait for Early Plant Vigor
Source: Front Plant Sci. 2017 Mar 28;8:420. doi: 10.3389/fpls.2017.00420 (PMC5368237; doi:10.3389/fpls.2017.00420)
Supplement: Supplementary file 5 [file Image_2.PDF]

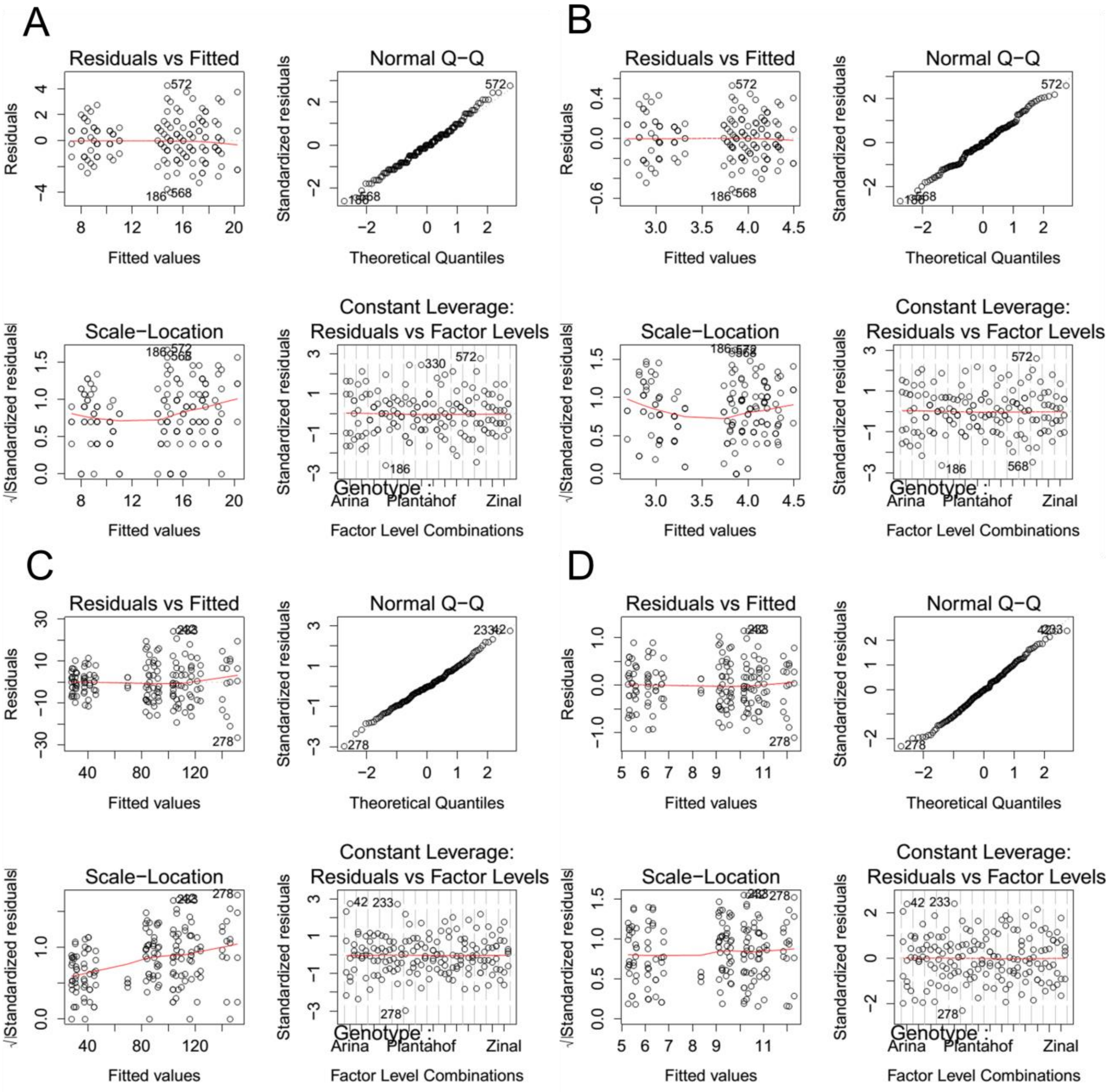

Supplementary Figure 2: Residual plots from two factorial analysis of variance model for (A) and (B) axial root number and (C) and (D) lateral root number (A) and (C) before square root transformation and (B) and (D) after square root transformation (n=4).
